# The aquatic animals’ transcriptome resource for comparative functional analysis

**DOI:** 10.1186/s12864-018-4463-x

**Published:** 2018-05-09

**Authors:** Chih-Hung Chou, Hsi-Yuan Huang, Wei-Chih Huang, Sheng-Da Hsu, Chung-Der Hsiao, Chia-Yu Liu, Yu-Hung Chen, Yu-Chen Liu, Wei-Yun Huang, Meng-Lin Lee, Yi-Chang Chen, Hsien-Da Huang

**Affiliations:** 1 Institute of Bioinformatics and Systems Biology, National Chiao Tung University, Hsinchu, 300 Taiwan; 2 Department of Biological Science and Technology, National Chiao Tung University, Hsinchu, 300 Taiwan; 3 Department of Bioscience Technology, Chung Yuan Christian University, Chungli, 320 Taiwan; 4 Institute of Molecular Medicine and Bioengineering, National Chiao Tung University, Hsinchu, 300 Taiwan

## Abstract

**Background:**

Aquatic animals have great economic and ecological importance. Among them, non-model organisms have been studied regarding eco-toxicity, stress biology, and environmental adaptation. Due to recent advances in next-generation sequencing techniques, large amounts of RNA-seq data for aquatic animals are publicly available. However, currently there is no comprehensive resource exist for the analysis, unification, and integration of these datasets. This study utilizes computational approaches to build a new resource of transcriptomic maps for aquatic animals. This aquatic animal transcriptome map database dbATM provides de novo assembly of transcriptome, gene annotation and comparative analysis of more than twenty aquatic organisms without draft genome.

**Results:**

To improve the assembly quality, three computational tools (Trinity, Oases and SOAPdenovo-Trans) were employed to enhance individual transcriptome assembly, and CAP3 and CD-HIT-EST software were then used to merge these three assembled transcriptomes. In addition, functional annotation analysis provides valuable clues to gene characteristics, including full-length transcript coding regions, conserved domains, gene ontology and KEGG pathways. Furthermore, all aquatic animal genes are essential for comparative genomics tasks such as constructing homologous gene groups and blast databases and phylogenetic analysis.

**Conclusion:**

In conclusion, we establish a resource for non model organism aquatic animals, which is great economic and ecological importance and provide transcriptomic information including functional annotation and comparative transcriptome analysis. The database is now publically accessible through the URL http://dbATM.mbc.nctu.edu.tw/.

**Electronic supplementary material:**

The online version of this article (10.1186/s12864-018-4463-x) contains supplementary material, which is available to authorized users.

## Background

Aquatic animals have significant economic benefits for humans and also play key roles in the development of medical applications [1, 2]. Aquaculture transforms natural aquatic resources such as fish, shrimp, and mussels into socially-valued commodities [3]. Chemicals derived from aquatic organisms are used to develop pharmaceutical compounds with important clinical applications [2]. In most of the aquaculture industries, prophylactic antibiotics have been used to prevent bacterial and virus infections [4].

To investigate the genomic resource of aquatic animals, next generation sequencing (NGS) approach has been adapted to uncover new genes and biological mechanisms. To effectively increase the our knowledge on aquatic animal gene discovery at mRNA level, RNA-seq has been applied to several important aquaculture species such as, *Plecoglossus altivelis* [5], *Cyprinus carpio* [6], *Penaeus monodon* [7], *Hyriopsis cumingii* [8], *Mytilus galloprovincialis* [9], and *Anguilla anguilla* [10]. To gain insights into the immunogenetics and immune response system, RNA-seq has been successfully adapting to study the host defense gene activities against bacteria and viruses [11]. *Clupea pallasii*, *Fundulus grandis*, *Pandalus latirostri*, etc. [12–15], have recently come under significant additional anthropogenic and environmental pressure, and SNPs and a set of genes associated with immune function in these species have been identified as providing high differentiation between two regions, and this insight can be applied in developing ecological monitoring systems. Evolutionary research on species including *Sinocyclocheilus angustiporus* (a comparative investigation between surface and cave species) revealed reduced expression of a series of visual photo-transduction and retinal disease-related genes in cave-dwelling species [16]. *Astyanax mexicanus* was also used to clarify heritable genetic changes governing adaptation to cave environments [17]. Beyond NGS transcriptomic data analysis, the MitoFish database focuses on fish mitochondrial genome annotation to explore a new resource for resolving fish phylogenies and identifying new fish species [18].

NGS technology has emerged as a powerful tool to illustrate the blueprints of novel species. Whole genome sequencing (WGS) and RNA sequencing (RNA-seq) can be used to investigate the individual differences, evolution and gene function [19, 20]. However, whole genome sequencing is more costly in terms of expense and computing resources than RNA-seq, and cannot obtain comprehensive information for transcriptomes directly by gene prediction. A better strategy is using RNA-seq to elucidate the molecular basis of biological functions [21]. Given the economical importance of these aquatic animals, a comprehensive resource of transcriptomes should be establsihed despite the lack of draft genomes of these non model organisms.

RNA-seq technology pave the way for transcriptome data investigation. With the techniques, transcripts can not only be detected in accordance with the reference genome but also can be used for de novo the assembly of RNA-seq reads to discover genes and profile their expression in organisms without a reference genome [22–24]. However, even if these data are deposited in the NCBI Sequence Read Archive (SRA) [25] which provides high-throughput unassembled and un-annotated raw NGS reads for more than 1000 Terabase pairs, public access is not provided to allow for searches of the well-annotated data. To address this problem, we have created a queryable database from the annotated transcriptomic database, called dbATM (http://dbATM.mbc.nctu.edu.tw). A similar approach was reported for ASGARD [26], which collects the annotated transcriptomes from three model arthropod species and NHPRTR [21], focusing on non-human primate NGS transcriptomic sequencing and analysis. dbATM is an easily-accessible database which integrates functional analysis applications.

**Fig. 1:**
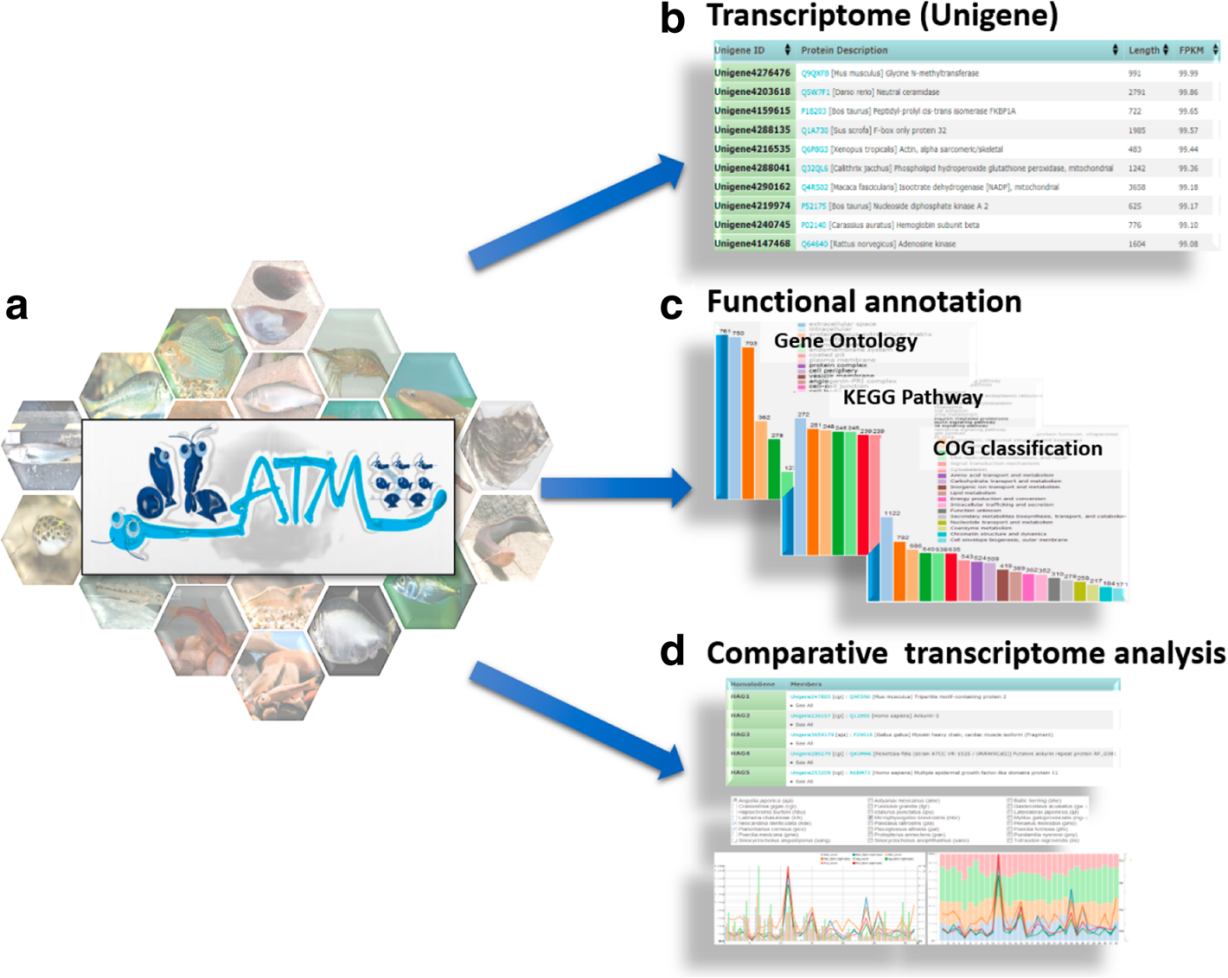
Aquatic animal transcriptomes maps database. **a** The database collects RNA-seq data for more than 20 different aquatic animals. The database consists of three parts: **b** Detailed information for individual genes such as gene name and description, length, expression (FPKM), and sequence. **c** Functional annotation of all gene groups in individual species. **d** Evolutionary studies of all species transcriptomic data by constructing a comparative analysis system for homologous genes

Aquatic animals have high economic value and also play important roles in the monitoring of environmental pollution in ecosystems. Aquatic animals’ related studies have seen exponentially increased in recent years (Additional file 1: Figure S1). Many studies have used NGS approaches to investigate genomic diversity among aquatic animals at genomic and transcriptomic levels. However, these studies largely focus on single species, and no databases exist so far to facilitate homologous gene mining among multiple aquatic species. In this study, we aim to establish an integrated aquatic animal transcriptomic database to facilitate studies in the fields of genomics, evolution, and phylogeny. We initially performed whole organism RNA-seq on four aquatic animals and collected RNA-seq data for eighteen species from the public domain. Reference genome free RNA-seq data of aquatic animals download from the three common NGS reads database, NCBI Sequence Read Archive (SRA), European Nucleotide Archive (ENA), and DDBJ Sequence Read Archive (DRA). Then, a computational pipeline was developed to annotate these NGS transcriptomes. Finally, all data were summarized and provided in the dbATM database. Figure 1 illustrates how the database can be used for mining transcriptomics, functions such Gene Ontology and KEGG pathways and comparative analysis of aquatic animals. The dbATM collects transcriptomes for twenty-two aquatic animals’ transcriptomes and provides an invaluable tool for homologous evolution and evo-devo studies.

## Results

### Overview of dbATM content

**Fig. 2:**
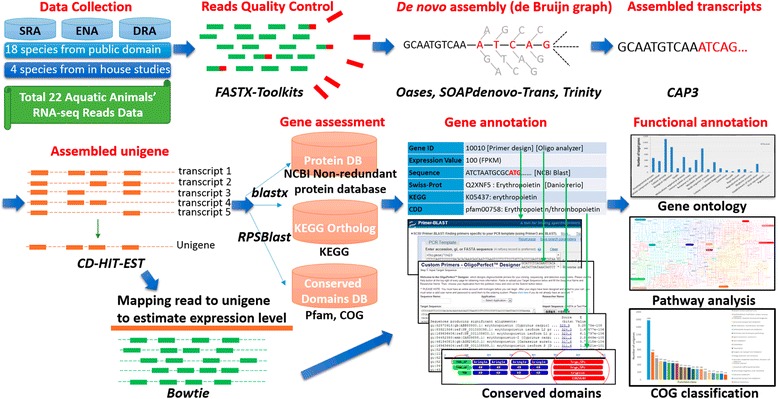
dbATM database system and analysis. First, publicly available RNA-seq data were collected and combined with additional data produced for this study. Next, the NGS raw data were trimmed and filtered to remove low quality reads. Third, NGS reads were assembled based on the de Bruijn graphs and clustered into transcripts or unigenes. Finally, assembled reads were represented to genes and gene functions by BLAST against external database

**Fig. 3:**
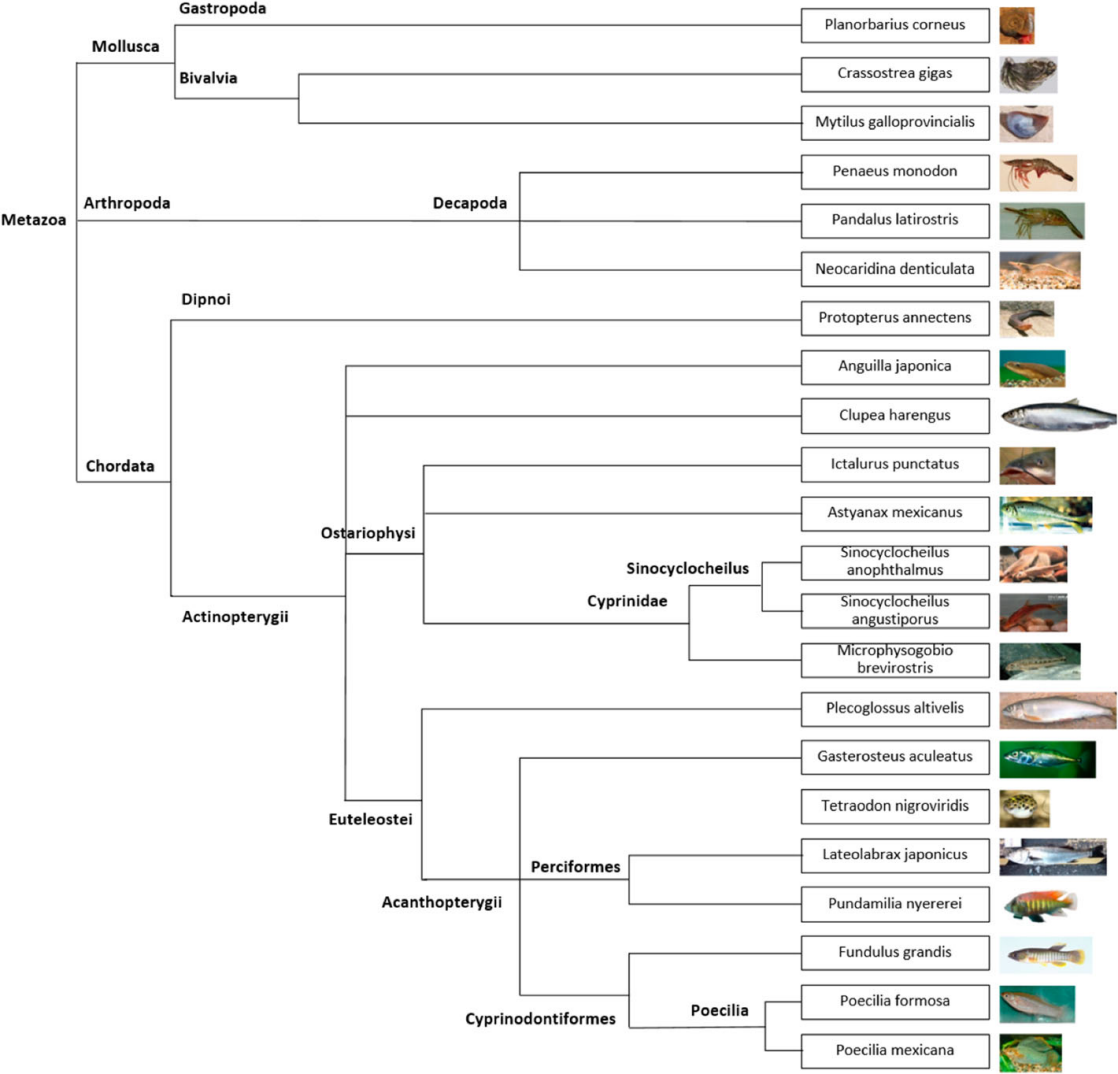
Species in dbATM and their biological classification. We categorize organisms into sub-groups and represent their rough evolutionary distances for further evolutionary and phylogenetic investigation

Additional file 1: Table S1 displays all aquatic animal RNA-seq data sources and original data types. Following systematic analysis (Fig. 2), the individual species information and comparative analysis can be accessed from dbATM. Table 1 shows the species annotation of the transcripts, unigenes, proteins, and homologous genes. To facilitate evolutionary studies and comparative analysis across the different aquatic animal species, a taxonomic tree was constructed according to biological classification (Fig. 3), for shellfish, shrimp, and fish from invertebrate to vertebrate.

**Table 1:** Gene annotation statistics in dbATM

Species	No. of transcripts	No. of proteins	No. of UniGene	No. of homologous genes
*Anguilla japonica*	99,696	38,815	13,600	9893
*Astyanax mexicanus*	59,723	23,085	10,443	7838
*Clupea harengus*	28,360	15,975	7713	5773
*Crassostrea gigas*	47,472	33,491	1149	377
*Fundulus grandis*	32,405	17,620	7388	4707
*Gasterosteus aculeatus*	77,510	43,170	14,035	6394
*Ictalurus punctatus*	57,435	28,850	13,400	10,452
*Lateolabrax japonicus*	27,075	15,130	6502	4227
*Microphysogo biobrevirostris*	50,827	31,923	14,839	11,295
*Mytilus galloprovincialis*	10,738	5944	815	141
*Neocaridina denticulate*	59,513	23,033	6593	1406
*Pandalus latirostris*	39,062	11,894	4127	1065
*Penaeus monodon*	36,979	14,740	4796	1162
*Planorbarius corneus*	61,364	20,951	3802	1004
*Plecoglossus altivelis*	43,524	22,836	9115	6544
*Poecilia formosa*	47,579	22,728	8271	3836
*Poecilia mexicana*	39,598	27,485	10,322	6423
*Protopterus annectens*	26,187	14,498	6050	2463
*Pundamilia nyererei*	19,205	13,762	5287	2749
*Sinocyclocheilus angustiporus*	51,104	41,391	11,505	8168
*Sinocyclocheilus anophthalmus*	81,704	48,526	13,583	10,452
*Tetraodon nigroviridis*	53,394	32,410	11,781	4238
Total	1,023,379	533,127	185,166	19,363^a^

^a^The total number of homologous gene groups show here is the real homologous gene groups in the database. Not the summation from individual species

### Web interface

**Fig. 4:**
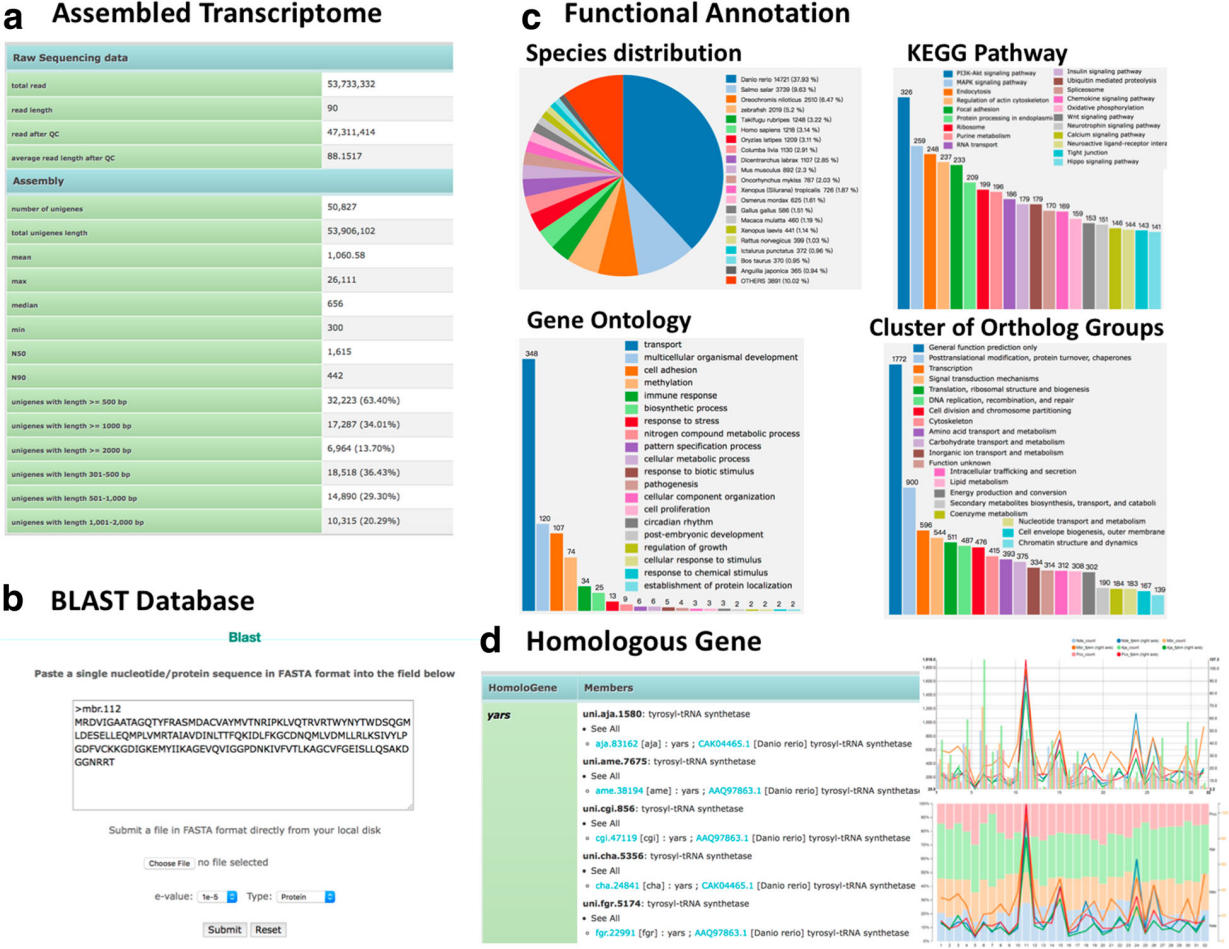
dbATM web interface. The dbATM provides various query interfaces and graphical visualizations to facilitate access to aquatic transcriptomic data. **a** Summary table of the reads, assembly quality, and annotation statistics. **b** A web interface to facilitate unigene mining by entering either sequence or keyword. **c** The functional annotation interface including annotated species distribution, KEGG pathway, Gene Ontology, and cluster of ortholog groups. **d** The homologous gene search and browsing interface for comparative analysis

The dbATM provides various query interfaces and graphical visualization pages to access to aquatic transcriptome data (Fig. 4). The summary table shows species information including scientific name (or common name), followed by total sequencing read size (in bp), sequencing platform, type of read sequences, number of reads and a link to NCBI SRA and NCBI Taxonomy and Wikipedia. Several browser functions were designed to allow users to obtain transcriptomic information. Users can browse by species, range of gene expression in FPKM, gene length, Gene Symbol, and Gene Ontology [27] based on homologous evidence, KEGG orthology pathway [28], COG [29] and Pfam [30]. An introduction page presents assembled transcriptomic information for each species, including statistics for raw sequence data, assembly quality and annotated genes (Fig. 4a, c). The embedded NCBI BLAST page allows users to input sequences or upload a text file containing sequences in FASTA format to search all genes sequences in the dbATM based on nucleotide or protein similarity (Fig. 4b). dbATM also allows users to browse homologous genes in homologous groups (Fig. 4d), and provides an interface for comparing KEGG pathway mapping results with the average FPKM of genes for a given pathway in different species, allowing users to select a maximum of 10 species for expressional profiling comparison.

### Investigation of aquatic animals’ homologous genes

Most current transcriptomic studies focus on single species, and these “standalone” transcriptomic databases do not allow for the exploration of homologous gene identities and their relative expression levels. To overcome this constraint, we provide a novel function to comparatively analyze potential homologous genes among 22 aquatic animal species. In total, 19,369 homologous genes were identified by OrthoMCL [31] and their gene name. Using homologous search function in dbATM, we can discover that several homologous genes (like *yars*, *cars*, *nop14*, *acsbg2*, *trip12*, *rab3gap2*, and *herc2*) are distributed from invertebrates (mollusca and arthropoda) to vertebrates (ostariophysi, euteleostei, actinopterygii, and chordate). The results shown that the essential genes such as the tRNA synthetase *yars* and *cars*, are evolutionary conserved. We also found there 21 homologous genes shared across at least 17 species: *yars, cars, nop14, acsbg2, trip12, rab3gap2, herc2, ddost, vwa8, cdk11b, ascc3, aplp2, loc563777, nup98, nup188, sdad1, nup205, dlat, acadm, rtn4ip1, kansl3*. The number of homologous genes in each clade were shown in Table 2. The homologous gene lists in each clade were shown in Additional file1: Table S2. Information on homologous genes across different animal phyla will provide good material for future evo-devo studies.

**Table 2:** Homologous genes statistics in each clade

	*Mollusca* ^a^	*Arthropoda* ^b^	*Ostariophysi* ^c^	*Euteleostei* ^d^	*Actinopterygii* ^e^	*Chordate* ^f^
Numbers	27	582	3027	85	19	8

^a^*Mollusca* clade: *Planorbarius corneus, Crassostrea gigas, Mytilus galloprovincialis*

^b^*Arthropoda* clade: *Penaeus monodon, Pandalus latirostris, Neocaridina denticulate*

^c^*Ostariophysi* clade: *Ictalurus punctatus, Astyanax mexicanus, Sinocyclocheilus angustiporus, Sinocyclocheilus anophthalmus, Microphysogo biobrevirostris*

^d^*Euteleostei* clade: *Plecoglossus altivelis, Gasterosteus aculeatus, Tetraodon nigroviridis, Lateolabrax japonicas, Pundamilia nyererei, Fundulus grandis, Poecilia Formosa, Poecilia mexicana*

^e^*Actinopterygii* clade: *Ictalurus punctatus, Astyanax mexicanus, Sinocyclocheilus angustiporus, Sinocyclocheilus anophthalmus, Microphysogo biobrevirostris, Plecoglossus altivelis, Gasterosteus aculeatus, Tetraodon nigroviridis, Lateolabrax japonicas, Pundamilia nyererei, Fundulus grandis, Poecilia Formosa, Poecilia Mexicana. Anguilla japonica, Clupea harengus*

^f^*Chordate* clade: *Ictalurus punctatus, Astyanax mexicanus, Sinocyclocheilus angustiporus, Sinocyclocheilus anophthalmus, Microphysogo biobrevirostris, Plecoglossus altivelis, Gasterosteus aculeatus, Tetraodon nigroviridis, Lateolabrax japonicas, Pundamilia nyererei, Fundulus grandis, Poecilia formosa, Poecilia mexicana. Anguilla japonica, Clupea harengus, Protopterus annectens*

### Investigation of gene expression profiles

**Fig. 5:**
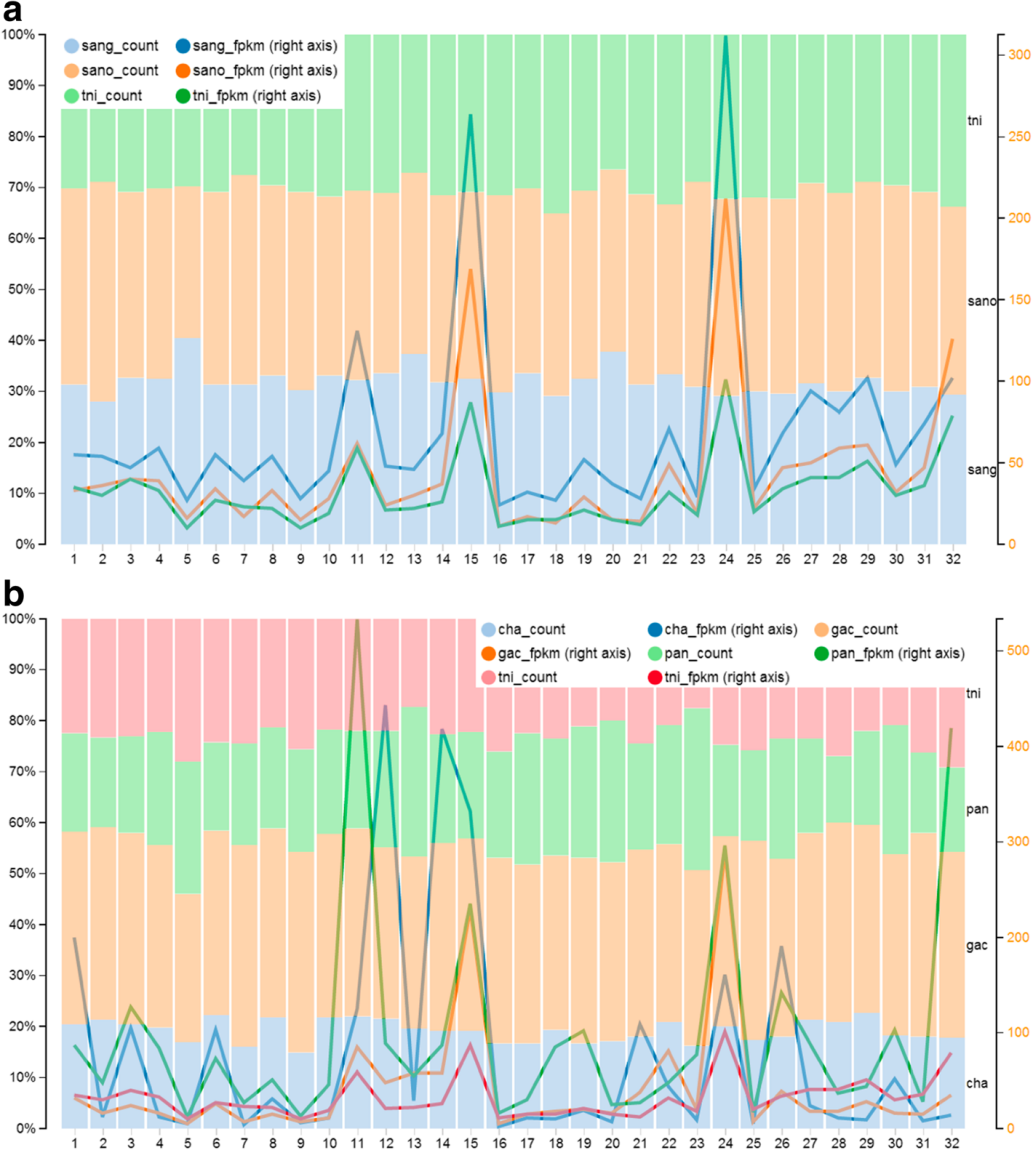
Gene expression profiles panel. The dbATM provides a function for comparative analysis of different tissues, conditions or species. **a** All three species samples are taken from the brain, presenting similar expression profile trends. **b** All four species are taken from different tissue, showing distinct expression profiles. Abbreviation code for species: *cha, Clupea harengus*; gac, *Gasterosteus aculeatus;* pan, *Protopterus annectens;* sang, *Sinocyclocheilus angustiporus;* sano, *Sinocyclocheilus anophthalmus; tni, Tetraodon nigroviridis*

To profile the gene expression level among different aquatic animals, we compare the gene expression value (displayed by FPKM) across different KEGG pathway categories. Results show the gene expression profile of a particular KEGG pathway stands out in some species. Distinct gene expressional profiling provides a good entry point to compare the gene expression level across different species or different tissue/organs among the same species. For example, the condition of the three species *Sinocyclocheilus angustiporus*, *Sinocyclocheilus anophthalmus*, and *Tetraodon nigroviridis* are from the brain tissue, and show similar expression profile trends (Fig. 5a). However, tissue for another four different condition species *Clupea harengus*, *Gasterosteus aculeatus, Protopterus annectens*, *Tetraodon nigroviridis* are whole organism, muscle, liver, and brain, respectively. The expression profiles panel of the four conditions (Fig. 5b) demonstrate quite shock and distinct.

## Discussion

We present a new systematic approach for de novo RNA-seq dataset analysis and annotation including improvement of de novo assembly quality and construct a new resource for the transcriptomic map aquatic animals for evolution and phylogenetic study. The system flow is illustrated in Fig. 2 and further detail is provided in the Materials and Methods section. First, we optimized the de novo assembly pipeline of RNA-seq data [32] by combining Oases, SOAPdenovo-Trans, and Trinity assemblers. To obtain complete transcriptomes from various assemblers [33, 34], we used the CD-HIT-EST cluster tool to merge Oases, SOAPdenovo-Trans, and Trinity assembling results. This combinational approach significantly reduce the transcript number and keeping a representative sequence for each unigene. Second, the gene annotation by BLASTX shows that some transcripts are mapped to the same gene or gene family. By following NCBI UniGene approach, we clustered the same gene to a group to reduce redundancy. Third, to facilitate the use of transcriptomes data for evolution and phylogenetic investigations, unigenes derived from all collected species were used to construct a homologous gene group. Besides, we provided the nucleotide (unigene) and protein sequence of all the 22 species on “Download Page”. The sequence data may apply the machine learning approach that could extract new features to develop gene prediction tools for aquatic animals. Finally, we develop a comparative analysis system by incorporating KEGG pathway analysis and gene expression profiles. This system assesses species and conditional diversity using the gene expression profile in specific biological pathways. dbATM not only improves de novo assembly quality of RNA-seq data but also constructs a database of the homologous genes of aquatic animals to allow for comparative study.

## Conclusions

dbATM is the first database to contain comprehensive transcriptomes annotations for more than 20 aquatic animals. The user-friendly web interface allows public access to valuable information for transcriptome assembly of each organism. The newly constructed homologous genes database and comparative analysis system also give aquatic researchers insights into the fields of eco-toxicity, animal physiology, comparative genomics and phylogenetic. dbATM also serves as an important repository for the aquatic animal transcriptomes by analyzing RNA-seq data. The assembly gene information provided could be a potentially valuable resource for designing expression microarrays to detect gene expression profiles across many conditions, thus facilitating studies in ecology and molecular biology. Given recent advances in NGS technologies, more non-model aquatic animal RNA-seq experiments will be deposited in the public domain.

## Methods

### System overview

First, RNA-seq data for 18 species of aquatic animals that were not well-annotated were collected from public resources such as SRA, ENA and DRA (the data from Illumina paired-end NGS sequencing platform were collected), with an additional four datasets produced for this study (Fig. 3 and Additional file 1: Table S1). Second, the quality of the raw reads from NGS was validated to trim and filter low quality reads. Third, the trimmed reads were then assembled through the designed combinational approach. Finally, genes functionally annotated by BLAST were compared against external databases (Fig. 3). All of the annotation results were soundly provided in the dbATM database.

### RNA-seq dataset collection

We collected eighteen RNA-seq datasets from the NCBI Sequence Read Archive (SRA), European Nucleotide Archive (ENA), and DDBJ Sequence Read Archive (DRA). RNA-seq data deposited to these datasets were generated from Illumina paired-end NGS sequencing platform. All RNA-seq data collected in dbATM were reference genome-free (including scaffolds or contig status) in the NCBI taxonomy database. We also performed RNA-seq on four additional aquatic species. Additional file 1: Table S1 present detailed information for the 22 aquatic animal species profiled in our dbATM database.

### Cleaning NGS read data

The NGS raw data for the RNA-seq in SRA format were converted to FASTQ format using the SRA-Toolkit v2.2.2 [35] and the FASTQ format reads were cleaned to increase read quality by FASTX-Toolkit v0.0.13 (http://hannonlab.cshl.edu/fastx_toolkit/). First, reads with adapters were removed. Second, the sequencing reads were scanned for quality at the reads tails. If the Phred quality scores were below 20 at the nucleotide end, the nucleotide sequences were removed. Third, if a read’s average Phred quality score was below 20, the read was discarded. Fourth, reads were discarded if their length was less than 70% nucleotides after tail trimming and reads filtering. Finally, for paired-end datasets, if one paired read had been removed in a previous step, the other read was also removed to synchronize the read pairs.

### De novo assembly

To ensure more complete assembly, we used three de novo assembly tools: Oases (v0.2.08, requiring Velvet v1.2.09) [36, 37], SOAPdenovo-Trans (release 1.02) [38], and Trinity (release 2013–02-25) [24] that based on the de Bruijn graphs. Previous reports suggest that coverage in transcriptomes assembly is not uniform. Higher *k*-mer lengths could obtain good quality assemblies for highly expressed transcripts. Lower *k*-mer lengths are used for poorly expressed transcripts [39]. This has been experimentally verified in model organisms [40]. We used multiple *k*-mer assemblies in Oases and SOAP-denovoTrans (for all species, *k* = 21, 29, 37, 45, 53, and 61, except for read lengths in *Fundulus grandis* smaller than 60, where *k* = 21, 27, 33, 39, 45, and 51). The insertion length of paired-end RNA-seq data was estimated using the observed-insert-length.pl program (included in the Oases package). Trinity was applied with default parameters. After the first step assembly in three assembler, we selected high read coverage and relatively long length as the representative transcript and conducted further assembly using CAP3 Version Date: 2007–10-15 [41] with an overlap length cutoff of 200 and overlap percent identity cutoff of 99 (−o 200 -p 99) [32]. To obtain comprehensive transcriptomes from various assemblers [33, 34], we used the CD-HIT-EST v4.6 [42] cluster tool with a sequence identity cutoff of 90% to merge results from Oases, SOAPdenovo-Trans, and Trinity.

### Abundance estimation

To determine the expression level in each species, we calculated the relative abundance of previously assembled results by RSEM (v1.2.3) [43] requiring bowtie (v1.0.0) [44] to accurately quantify transcript expression with or without reference genomes. The relative abundances of transcripts were measured in unit of normalized reads count aligned on de novo assembly, FPKM (Fragments Per Kilobase of transcript per Million mapped reads) [45]. After de novo assembly and expression calculation, we select FPKM > = 1 and gene length > = 300 as a cutoff for further analysis such as functional annotation and comparative analysis.

### Functional annotation

BLASTX (v2.2.28+) [46] with E-value cutoff of 1.0e-5 were used to homology search the NCBI non-redundant protein database (nr, v2012–11-19) for the assembled genes. The best blast hit protein was annotated to the representative gene and protein name. The gene annotation by BLASTX indicates that some transcripts were mapped to the same gene or gene family. To reduce redundancy, we used the NCBI UniGene approach which clusters the same gene to a group. The results for the BLASTX annotation genes were mapped to the Gene Ontology database. We also extracted the open reading frames (ORFs) from all genes according to the best BLASTX match hit results. To identify genes involved in KEGG pathway, we used the KEGG Automatic Annotation Server, KAAS [47]. Finally, we identified the putative protein domains on translated protein sequences extracted from genes by aligning to CDD (conserved domain and protein classification) [48] using RPS-BLAST.

### Comparative analysis

Based on a comparative analysis, different species were grouped into three main categories. First, we used OrthoMCL [31] with an E-value cutoff of 1.0e-10 [49] to perform a similarity search orthologous by computational approach, and then defined the orthologous groups using the representative gene name or description, and called each group a homologous gene group, indicating that the different species shared a common ancestor. Second, we compared the abundant gene expressions among the different species using the functional annotation KEGG results and the D3: Data-Driven Documents [50] to provide enhanced graphical representations. Considerable amounts of annotation information was obtained from the KEGG pathway category [28] with average of gene expressions (FPKM) in different species. In Fig. 5, the bar is the read count aligned to each KEGG pathway category, and the line is the average FPKM of the genes mapped to this pathway. In the KEGG pathway category, we excluded Human Diseases, Drug Development, and Global Maps from the metabolic category. The graph visualization of comparative analysis represents the gene expression profile in different tissues, conditions or species using KEGG pathway. For example, the Fig. 5a illustrates the samples from the brain, so the expression profile trends (gene count and average FPKM) are similar. In contrast, the Fig. 5b displays the samples from different tissue, so the expression profile trends are distinct. Third, a web interface of BLAST against dbATM to allow the user using blastp/blastn to search protein/nucleotide data by pasting the query sequence or by submitting a file in FASTA format.

## Additional file

Additional file 1: Figure S1. The cumulative publications of related articles of aquatic animal in PubMed. **Table S1.** RNA-seq datasets from twenty-two aquatic animals were analyzed in dbATM. **Table S2.** Statistics of homologous genes in each clade and their gene lists. (PDF 280 kb)

## Abbreviations

COG: Clusters of Orthologous Groups
DRA: DDBJ Sequence Read Archive
ENA: European Nucleotide Archive
FPKM: Fragments per kilobase of transcript per million mapped fragments
KEGG: Kyoto Encyclopedia of Genes and Genomes
NGS: Next-generation sequence
RNA-seq: RNA sequencing
SRA: Sequence Read Archive
