# Supplementary material for: The aquatic animals’ transcriptome resource for comparative functional analysis
Source: BMC Genomics. 2018 May 9;19(Suppl 2):103. doi: 10.1186/s12864-018-4463-x (PMC5954267; doi:10.1186/s12864-018-4463-x)
Supplement: Supplementary file 1 — The cumulative publications of related articles of aquatic animal in PubMed. Table S1. RNA-seq datasets from twenty-two aquatic animals were analyzed in dbATM. Table S2. Statistics of homologous genes in each clade and their gene lists. (PDF 280 kb) [file 12864_2018_4463_MOESM1_ESM.pdf]

Additional files 1

Supplementary Figures

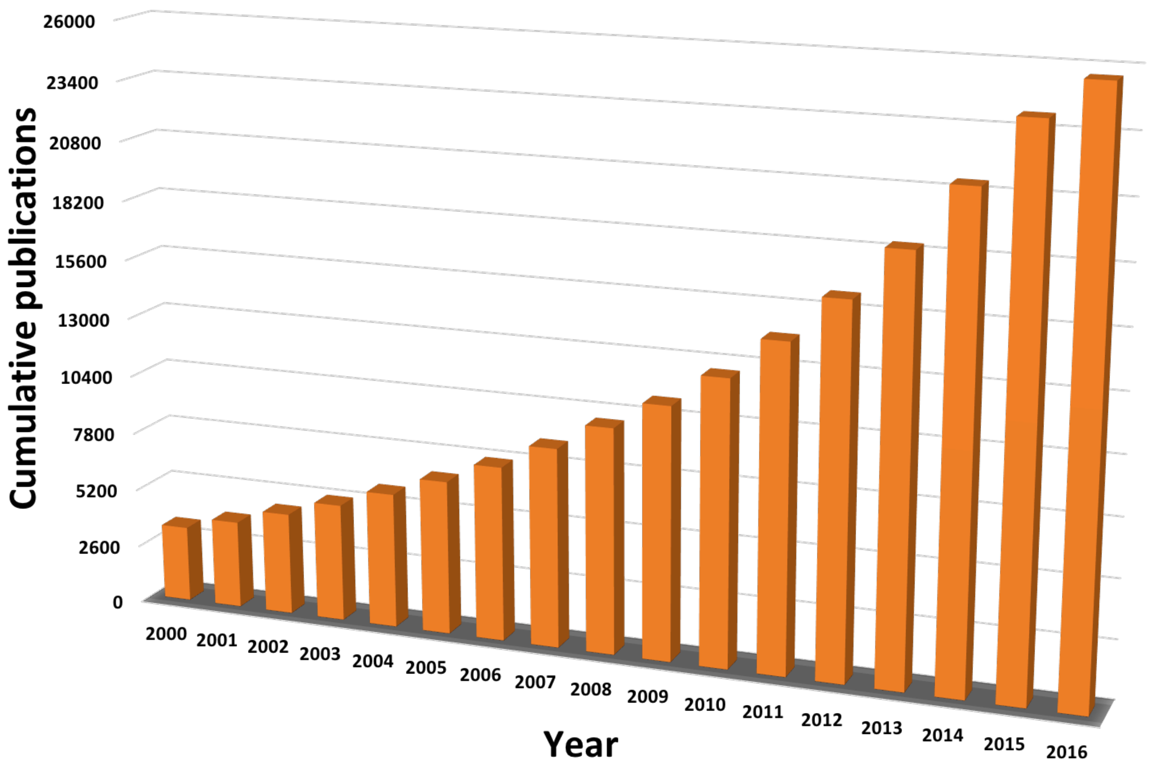

Figure S1. The cumulative publications of related articles of aquatic animal in PubMed.

Supplementary Tables

Table S1. RNA-seq datasets from twenty-two aquatic animals were analyzed in dbATM.

Species	No. of Bases (Gbps)	Platform	Read length	Source
[a] ***Anguilla japonica***	8.61 G	Illumina HiSeq 2000	PE, 2x101	SRR1185326
Astyanax mexicanus	8.88 G	Illumina HiSeq 2000	PE, 2x100	SRR688577
Clupea harengus	11.72 G	Illumina HiSeq 2000	PE, 2x101	SRR611605
Crassostrea gigas	2.86 G	Illumina HiSeq 2000	PE, 2x93	SRR497890
Fundulus grandis	5.00G	Illumina Genome Analyzer IIx	PE, 2x60	SRR504405
Gasterosteus aculeatus	3.20 G	Illumina HiSeq 2000	PE, 2x101	SRR528545
Ictalurus punctatus	4.96 G	Illumina HiSeq 2000	PE, 2x101	SRR392743
Lateolabrax japonicas	2.59 G	Illumina Genome Analyzer II	PE, 2x75	SRR536341
[a] ***Microphysogobio brevirostris***	4.84G	Illumina HiSeq 2000	PE, 2x90	SRR1185341
Mytilus galloprovincialis	1.37G	Illumina Genome Analyzer II	PE, 2x100	SRR442035
[a] ***Neocaridina denticulate***	6.06G	Illumina HiSeq 2000	PE, 2x101	SRR118532
Pandalus latirostris	4.74G	Illumina Genome Analyzer II	PE, 2x76	DRR001119
Penaeus monodon	5.60G	Illumina Genome Analyzer II	PE, 2x72	SRR388221
[a] ***Planorbarius corneus***	5.66G	Illumina HiSeq 2000	PE, 2x101	SRR1185333
Plecoglossus altivelis	4.48G	Illumina HiSeq 2000	PE, 2x90	SRR363084
Poecilia formosa	5.72G	Illumina HiSeq 2000	PE, 2x100	SRR629501
Poecilia mexicana	1.63G	Illumina HiSeq 2000	PE, 2x101	SRR534470
Protopterus annectens	1.94G	Illumina HiSeq 2000	PE, 2x76	SRR505724
Pundamilia nyererei	2.10G	Illumina HiSeq 2000	PE, 2x76	SRR445779
Sinocyclocheilus angustiporus	1.10G	Illumina Genome Analyzer II	PE, 2x74	SRR788094
Sinocyclocheilus anophthalmus	2.06G	Illumina Genome Analyzer II	PE, 2x74	SRR788095
Tetraodon nigroviridis	11.43G	Illumina HiSeq 2000	PE, 2x76	SRR579565
**22 Species**	106.55 G			

[a] The RNA sequencing data sets generated in this study.

Table S2. Statistics of homologous genes in each clade and their gene lists.

Clade (No. of Genes)	Gene Lists
Mollusca **(27)**	*arhgap17b, as3mt, wdr21, zgc:175171, ttll12, fam102bb, plekhm3, mplkip, nme6, klhl38a, reep3l, loc101077359, slc25a47a, loc101078827, blvra, foxo3b, loc100703121, pkn3, loc101067704, ptgdsa, loc101163770, mab21l1, phum_phum086910, loc101066367, loc101064786, dhrs11b, loc100706188*
Arthropoda **(582)**	*cars, acsbg2, ddost, ascc3, aplp2, nup205, dlat, acadm, kansl3, gcn1l1, wipi2, ascc2, usp25, bsdc1, nsfl1c, cnot1, snrnp70, prpf39, ralgapa1, ranbp9, mkln1, pccb, aqr, snrnp200, copb2, dhx16, wdr11, aup1, nkrf, patl1, rufy2, rint1, smg5, nfatc3, vps33b, zgc:85858, bckdha, neo1, sppl3, psmd4a, osbpl9, fahd2a, dmgdh, pmpca, xrn2, rps15a, apc, atrx, kif16b, tcf7l2, ubap2l, ube4b, nckap1, smap1, ndrg2, mef2d, bms1l, kpnb1, fkbp8, loc101167943, nmt1a, smurf2, znf259, cyc1, zmat2, loc101167297, tusc3, iars, anxa6, samm50, tubgcp2, fkbp1b, gstcd, mesdc2, tbc1d23, fbxl4, abcc9, mfge8b, clasp1a, letm1, dlg5a, arl5a, sumo1, acadl, crebl2, emc10, moes, farsa, fnbp1, heg, loc100700623, loc101158698, clstn1, ptpn13, tbc1d16, cct8, s41a2, crot, npat, disp1, ankrd44, slc25a37, edem2, slc43a1b, trmt11, cpox, gtf3c1, 2a5e, si:ch211-225b11.4, gtf2a2, dohh, prrg4, ndrg3b, cn37, gnal, nosip, msmo1, nadka, ict1, ythdc1, tnfaip2a, thas, tipin, akap17a, nfasca, pcdh19, si:ch211-191j22.3, spen, hk2, loc566587, slc13a5a, loc101165302, ncoa3, ppp1cab, btd, mrto4, ldhba, reep3l, prickle1b, mis18a, pold1, dpy19l3, arhgdia, si:ch73-381f5.3, zgc:112962, tada2b, cmklr1, cygb2, loc100708444, cmss1, loc101167287, vir, loc100694931, mrp1, loc100709525, kntc1, smcr8, loc101165712, exo1, loc100693412, stard10, nek8, loc101169719, im:7140390, rayl, nudc1, bmf2, loc100135972, pax7b, tmem106a, snx22, loc100689736, loc101172479, sh2d3cb, loc100692541, si:ch1073-228b5.2, loc100701920, sias, wdr90, cacna1i, loc101170264, loc101162834, zgc:92598, cnpy1, fam49bb, pddc1, ccdc120, loc101169363, loc101174617, loc100695023, zgc:113425, rbpja, loc100712113, tcf7l1b, slc37a4, phum_phum349810, loc101067921, loc101079580, loc100697359, loc100004299, dnm1b, loc100691791, syt1a, gls, psmb1, kiaa1324, loc100694401, loc101172999, loc100150849, loc100534505, stxbp3, si:dkey-78l4.14, nemvedraft_v1g93294, ntrk3b, loc101067217, loc101170479, chrne, loc566697, mkrn4, cx32.2, vrk2, loc101173447, loc101175430, loc100702159, loc100707227, hs3st3b1a, loc100538228, loc101166998, phum_phum551470, loc562425, loc100014518, loc100694330, il1rapl1b, zgc:153049, wdr47b, chd6, dvirgj20269, f101b, gcdhl, rnf144ab, loc101062834, loc100691107, loc101074613, loc553528, loc565380, ldb3b, loc102092046, pdzd1, loc797545, loc101065804, si:ch211-260b17.8, thex1, loc101079666, loc100691626, ccdc149b, loc100701076, mmp16, loc101158432, nhlh2, gli2a, jmjd5, si:dkey-260c8.8, drd2b, slc2a11b, tph1a, loc101168203, loc101173830, pla2g12b, mgp, kif3c, lcorl, phospho1, gdf10b, zgc:194993, loc100697193, loc101079588, svil, loc101160300, loc100703466, loc101070223, syn2a, loc101167898, hs3st4, pitpnbl, loc100708771, mda5, loc101062051, trpm5, loc100695565, sh24a, aael_aael010795, rfxk, si:dkey-10c21.1, loc101069285, loc100693512, zgc:171459, aael_aael001936, loc100330817, loc101155516, phum_phum369800, rhof, loc101159255, loc101064440, loc799320, arhgef18a, loc100333406, cl043, loc101065367, filip1, kif5a, c1qt4, loc101066414, loc101173270, creld2, loc100691409, plp1a, loc101080243, loc101161201, loc101168874, loc100889615, gareml, loc101156712, iscw_iscw010383, enthd2, fglh2, loc100692206, polr3k, loc100184639, phum_phum163920, cbln2b, loc100000890, loc100710357, dyakge21178, faub, plg, loc100136217, loc101168686, itgb1bp1, myl2b, loc101166731, loc101164061, gtf2e2, zgc:63831, edar, agap_agap004559, hsh2d, iscw_iscw010384, loc101170105, cldn19, lmtk3, agap_agap012513, vang, arpp19a, loc101173897, daw1, loc100149559, loc100709044, phf21aa, c19orf66, loc100703013, loc100378244, loc100711593, loc101076835, iscw_iscw005899, scamp4, loc100330886, loc101162092, zgc:154169, loc101164844, phum_phum066650, loc100705002, fam203a, loc100696634, loc562098, loc100705095, dok6, trpc4apa, hadh, mgst2, loc101155248, gramd4, psmd8, loc101078033, loc101077354, tsnare1, hnf1bb, wls, loc556200, loc100709351, azl, neurod2, cd93, loc101168763, phum_phum378970, loc101066577, loc101068706, loc100692768, loc101072108, cox7a2a, pitg_07096, loc100333456, loc562156, si:dkey-261l7.2, psmb7, loc101072417, loc101071808, phum_phum600830, loc101074632, loc101164620, loc101169449, loc101164673, phum_phum022330, loc100698520, phum_phum232370, loc101174460, loc101078370, pmt, loc101161575, iscub, loc101075829, loc100710068, loc100703981, loc100377112, abcd3, stx12l, loc100706642, loc101070589, loc794616, tmbim1, loc101173173, kitlgb, ctsh, loc100378642, loc100710523, loc796439, loc101076896, lycat, mrps10, loc101063813, myl6b, npm2, loc100888931, loc101166823, scrt1a, loc101165025, loc100698833, aael_aael014408, si:ch211-173p18.3, loc101169132, loc101075113, loc100174884, loc100148595, loc100699090, loc100149007, im:6907928, loc101071846, aael_aael006619, loc101080167, loc101063722, loc101064354, loc101066532, noxo1a, loc101169692, loc100711918, prpf4ba, loc101061869, etgase, pcdh2g8, sdc, loc101161482, arl15, psa4, irgf1, h2afvb, loc101069044, cdpf1, loc100174885, loc101064751, loc101065446, xpp3, phum_phum127760, wdr44, loc100711269, f135a, si:dkey-61p9.6, loc100689926, loc101074645, nog3, parp4, nemvedraft_v1g98785, trerf1, loc100709367, mitfa, phum_phum037410, loc568267, loc101155642, fam166b, trim45, loc100708669, loc101068206, loc101170995, fto, rfa3, si:ch73-389b16.1, loc100707685, si:dkey-102f14.5, plcd1a, loc100689929, yeats2, loc100710533, loc101070714, ak7, loc101162199, loc101170686, zgc:172075, foxi3b, mms19, cnpy2, rd3, loc100708672, emid1, tcpd, aael_aael009869, agap_agap011820, cds1, loc100710158, loc100371366, loc100706587, pld1b, kcnh6, camk1db, loc100705737, iffo1, loc101071992, zgc:171844, oxr1b, tmem176, msna, myo6a, fdx1, loc101061194, loc101167959, psap, loc100708732, si:dkey-25o1.5, phum_phum106430, loc101065457, loc101073065, tmem86b, phum_phum308880, htra1, prdm5, sels, f13a, loc553495, sema3gb, loc100691183, kifc1, loc100151586, tfe3b, loc100002384, loc101066439, zgc:171531, loc101078876, loc101173313, loc568340, esco1, loc101065163, loc100194697, oat, lox5, odz3, loc101073668,*

	loc101077877, loc101166238, arpc4, loc101173940, si:dkey-51e6.1, ehd1a, loc101071819, zgc:112392, col9a3
***Ostariophysi* (3027)**	*yars, cars, nop14, acsbg2, trip12, rab3gap2, herc2, ddost, cdk11b, ascc3, loc563777, nup98, nup188, sdad1, dlat, rtn4ip1, kansl3, loc100304460, nfyc, srm, dync1h1, ddx54, wipi2, brpf1, p4hb, stau1, usp9, wrap73, ext2, usp25, sep15, skiv2l, rgp1, mtap, xpo6, zdhhc17, os9, aifm1, tnip1, bsdc1, thoc2, ivd, scp2a, snx14, csnk1a1, eif3d, zw10, rbbp6, atm, tln1, ift172, uvrag, ctr9, vps13a, ttc27, retsat, hectd3, rab3gap1, prcp, fn1, snrnp70, tsr1, mcmbp, snx2, xpo4, pepd, prpf39, adhfe1, sell, tex2, nolc1, scyl3, tcp1, sdha, xpot, opa1, atrn, brd7, mkln1, zgc:158234, pccb, elp3, ube2h, utp18, si:ch211-233a24.2, supt6h, rere, cct6a, sec13, clk2a, rtca, prpf8, ogt.1, copb2, rab2a, ip6k2, psmd2, narfl, clk4, dkc1, eif3g, plekhm2, dock7, armc8, dhx16, sept2, rspry1, spast, golga2, acadvl, spg7, loc100703757, morc2, lrpprc, abca5, phb, xrcc5, hat1, notch3, eif4g2a, cdc27, dennd5a, trmt2a, csk22, fryl, sqrdl, parp1, ahcy, golga4, ddx27, cdc5l, slc25a42, gapvd1, slu7, cabin1, abcc4, noc2l, adck3, nprl3, prps1b, pex5, sptlc1, exoc3, nkrf, mapk6, hbp1, nup85, ints3, ktn1, smarca2, pgm1, nelfa, nsd1, mgrn1b, agps, fkbp5, mettl13, vmp1, vps45, uba3, pmpcb, smarca4, med14, acox3, fubp3, sbf1, pds5a, tnpo3, dennd1a, sps2, pitrm1, poll, rexo1, fh, cpsf1, skiv2l2, nubp1, wsb1, scfd2, rint1, 1500011k16rik, sdf4, cdk9, apbb1, xpo7, pdcd6ip, bcl9l, rbm45, cwf19l2, ncstn, zswim8, slc25a20, ipo7, ngly1, edc4, ube2g2, eef2k, dhx38, optn, si:dkey-158b13.2, supt5h, zgc:63566, timm10, ap3d1, eefsec, chd1l, prkar2aa, cand1, cdc23, add1, gpcpd1, eepd1, tsc22d3, f8a1, nfatc3, aldh16a1, slka, exoc7, abca1b, sepsecs, dus3l, ptprb, atl3, gas6, pcm1, xrcc1, celf2, osbpl2b, zgc:113076, bre, vps33b, rpap3, zgc:158450, lig3, cnot10, usp36, zgc:162584, zgc:111986, si:dkey-13i19.8, uhrf1bp1l, plxnd1, wars, lrp6, nle1, pdcd11, xylb, phf8, eif2b4, tars, faf1, pbrm1, cmc2, romo1, nf1, trim23, tia1, ckap5, mcrs1, mocs1, bckdha, ctdspl2a, chm, mapkapk5, actn4, arf1l, klhl12, cox1, mfsd1, perp, dnm1l, pcf11, alg8, wdr3, srebf1, mtr_5g050970, vcp, rrp12, dgkaa, nbeal1, golga3, prpf3, rabgap1, loc100697653, sf3a1, sppl3, ikbkg, zgc:73111, ubtf, osbpl9, mios, cops3, fahd2a, loc558044, si:ch211-114c12.2, zc3h13, slc9a7, snx1a, hip1, cog4, sardh, lcp1, loc101071273, acy1, dpp3, hectd4, dmgdh, kpna4, zgc:63733, kdm5c, csde1, afg3l2, srp68, tubgcp5, kif1b, cluh, eif4e2, svip, kdm1a, uso1, tdh, smek2, men1, ptcd3, tbce, ddx6, ubc12, arl8ba, ppp4r1, dscr3, naa25, slc38a4, dctn4, ap1b1, slc27a1a, ralbp1, pgm2l1, isca1, xrn2, nsun5, lbr, ankfy1, vipas39, zgc:92107, asna1, vps25, tssc1, pikfyve, kpnb3, kdm6bb, smc3, pan2, sept6, eif3s10, chuk, erp44, prpf40a, aldh6a1, arid2, exoc5, smc4, acp2, ccpg1, sart3, asns, ttc17, trim3b, bap1, smc5, ubr1, wdr43, strip1, micu1, trappc11, fastk, nucb1, ppp5c, mgea5, rps6kb1b, atp9b, gmps, cul9, ddx46, pik3c2b, pnpla6, apc, enpp2, lnpep, ct011, map3k5, sel1l, loc100149834, qrich1, nono, prmt1, arhgap12b, tbc1d17, lmbrd2b, nedd1, exoc8, tecpr2, atp6v1c1a, abhd12, erbb2ip, klhdc4, mkrn1, zgc:158262, ttc21b, rad50, snx17, ap1s2, pcy2, chd4a, kansl2, dhtkd1, kif16b, rasa3, rnf14, hnrnpub, ino80, acads, erlin1, dnpep, gba, lamc1, tecrb, kcmf1, taf7, lsg1, zfr, fbln5, golga1, itgav, canx, fbxo25, tbca, nvl, aldh1l1, p4ha2, tcf7l2, reln, pcyox1, rfc4, mphosph8, sucla2, zgc:154077, loc100194703, fbxw5, ubap2l, ccdc22, ube4b, pwp1, taf1, kif3b, lrrfip2, nob1, dis3l, ahctf1, rngtt, cops2, aip, cox10, sesn1, dusp1, ocrl, magoh, fam199x, tchp, glsa, loc101079214, htatip, selj, tubgcp3, ilk, agla, fam96b, eif2d, ank3, mars, nckap1, numb, piga, vps39, foxk2, pex3, ap2b1, gusb, smap1, mdm4, fgl2, pdcd4a, hmgcs1, ufl1, fxr2, clptm1, lss, dyrk1b, slc7a10a, ano10a, gtf3c5, snw1, llr1, arl8a, cog5, kat2b, abcc5, ufm1, ctnnal1, dcaf5, loc101160968, noc3l, fn1b, nup107, loc101161406, sf3b1, mta2, tcf12, clcn7, znf384l, tmem68, prdm10, cpne1, selk, chid1, mef2d, srrt, pleca, cratb, rnls, gfm1, blmh, adck5, nup133, zswim5, uri1, cdc73, gpkow, cnot6l, bms1l, afmid, slc30a5, rev3l, aplp1, xrn1, oser1, calrl2, tgfbrap1, azin1b, rbm22, loc568087, galnt11, tmem181, ryk, snrpb, usp10, rpl23, synj1, usp2a, smarca5, mtor, sh3pxd2aa, mib2, dctn5, si:dkey-180p18.9, mettl7a, ddx1, nfat5, nup155, babam1, zgc:158619, loc100194728, pfas, ndufs1, man2a2, zgc:110816, taf2, slc12a9, ergic3, myh10, nup160, atad1b, thoc1, fam160b1, loc101167943, myof, aacs, rps27a, si:ch211-198a12.6, mri1, trappc10, parp2, prkcq, fbl, gfpt1, loc567075, mfn1b, loc101171598, cdc42bpb, ubac1, macf1, loc100170209, n4bp1, smg7, cpsf3l, sepp1a, si:dkey-159a18.7, prkcsh, gnptab, rock2a, slc20a1b, myh11a, anks1b, dnajc2, mccc2, slc25a36a, khsrp, fgd4a, polr1b, mpeg1, atp6v1ba, ubxn7, abcd4, gemin8, zgc:55582, ddi2, mgat2, tmem184c, gtf2h4, usp5, aars, abce1, dgcr2, osbpl1a, impdh2, si:dkey-98p3.1, pls3, pik3c3, use1, cyc1, fam214a, mgll, efr3bb, pdlim5b, yth2, eif4a2, pi4k2a, vps8, ppox, zmat2, lpar6a, exd2, nrd1, mtmr3, nkap, pole, vars2, dhx9, dcaf11, rqcd1, iars, hnrl1, rrn3, daam1b, osbpl5, eprs, eif3f, pdlim7, mybbp1a, ddx51, loc101163139, usp19, hinfp, ccdc6a, fam20b, ccdc9, psma8, dhx15, clpb, dym, ncoa4, asxl1, vps36, cmtr1, mrps5, arhgap32, btbd7, rabep1, prrc1, gpr108, cs061, pdcd2, zgc:175222, samhd1, smim14, zgc:112178, kif3a, traf7, wipi1, slc15a4, mvp, ssx2ip, vps11, pigs, pm20d1.2, galk1, mgat5, slc9a6a, ptprs, krit1, irf2bpl, usp8, xpnpep1, loc100706437, hmg20a, rfc5, qars, ctnna1, ei2be, fkbp1b, suclg2, hook3, zgc:101116, gstcd, fech, usp22, timm44, ap4s1, tmem64, arpc5b, abhd16a, mesdc2, flii, si:dkey-91i10.3, smyd5, ddx56, kpna3, setd7, fbxw8, oaz2, slit2, myo9a, c14orf159, ccm2, agpat9, mest, golga5, klhl15, tbc1d25, svila, zzz3, dusp8a, tmem248, pitpnc1, xpo1, loc100695567, atp6v0a2b, zgc:77375, map4k5, tsg101a, vat1, zgc:162967, taf12, tex10, loc556764, ostf1, ulk1a, dync1li2, nckipsd, zgc:154116, zgc:56039, slc1a4, dlst, mbd1, nxpe3, zdhhc14, ralba, cog6, tubgcp6, mib, zgc:86598, idh1, aidb, fbxl4, oxct1a, ddx31, mbnl3, ppm1g, bag6, atg7, usp1, sf3b2, garnl3, tmem251, pitpnm2, kiaa0930, hacl1, pptc7a, srp9, abcc9, fam63a, huwe1, diaph2, ufsp2, cln3, asmtl, cwc27, mfge8b, ptpn1, pros1, rab40c, trim32, ppm1h, rif1, rabggtb, aldh4a1, fcho2, atg9, pms1, pacs2, loc101078475, siae, sly1, psl2, sike, nsdhl, tpp1, atp6v0d1, cef10, slc35f6, acsl4a, si:dkey-5i3.5, abhd4, ist1, ankrd12, arhgap1, pbx4, fnbp1l, ptpn11a, kdm2aa, iqgap2, ap1g1, paics, wdr18, bhmt, wnk1, r3hdm1, si:busm1-180o5.3, neu1, g3bp2, loc100697419, ints8, setb, fbxo7, riok1, usp30, utp15, ubl3b, hivep2a, pum1, tsc22d1, larp6, rfwd2, mgt4a, actl6a, uggt1, cct4, dip2ba, phrf1, prkar1ab, dmtf1, dlg5a, cd97, dnajc19, fam134c, uap1l1, ipo8, snrpd1, khk, arl5a, pcsk7, slc30a9, gpr89, pkp4, cstf3, mpdz, si:dkey-46a10.3, agtpbp1, ptdss1, adam10a, birc6, spag9, brd8, loc101070072, dsp, loc101155599, wu:fb99d03, setd1a, adipor2, rcl1, usp32, stk11, tcp11l2, nek1, api5, ctdp1, col4a1, srsf1b, ago4, hnrnpm, ptdss2, yrk, tmem11, rbm5, coro7, rpap1, hps4, pex12, dennd2da, gldc, usp33, hsd17b8, nrbp1, psde, stom, tlk1b, hagh, ubr3, fktn, pdip3, sumo1, fbln1, zgc:56194, ppm1aa, thoc6, scpdh, gle1, jhdm1db, mysm1, tdrd3, rfc1, gclc, cbl, zgc:112350, taf6, ptbp1a, araf, hdac1, ltn1, ncoa2, loc101173374, add3a, setd2, kdm4ab, eltd1, ccdc47, rdx, glrx3, utp6,*

rnasekb, hspe1, smim12, wdr70, smox, fam73a, wdtc1, prex1, ehmt2, fam91a1, rgl1, dgcr8, nol10, vps35, vps9d1, loc100696349, setd3, tmem209, jmjd1c, loc101065346, usp6nl, mtmr6, slc35e1, mpzl1l, nnt, dirc2, dag1, mvk, axin2, brcc3, dnaja3a, gon4l, fam13b, tbp, vta1, sympk, hic1, pnkp, il-1racp, agap2, wdr26b, map2k7, enpp6, mef2aa, tesk2, trabd, mtss1l, fkbp1ab, loc555713, mark3, brd1, zfr2, gga1, brf1b, gopc, rbms1, dnajb9a, gigfy2, cdc26, loc100699880, setd5, ush1c, cyfip1, usp39, ola1, agap3, tprb, lats1, sema4ba, slc43a3b, dctn1a, gnl3, nudt19, fnta, ddx55, stag1, atf6b, kdm5ba, rad51c, nisch, rn139, nedd9, pgs1, uvssa, cecr1a, loc101168325, lats2, loc100704097, zfyve1, scamp1, erc1, pbx1a, vps13b, dll4, gse1, dnajc1, sesn2, galnt7, sdccag8, timm50, llgl2, moes, snx5, kiaa0907, gbe1, psmb2, nek9, ubtfl, ccnt2a, orc4, nasp, loc101071869, rnf24, dalrd3, rbm17, ddx28, ruvbl2, ppp1r10, rpia, sf3a3, slc25a43, wasf3, ogdha, apaf1, ubiad1, polh, prkag1, abl2, eif2s1b, net1, vps13d, acap2, ddx3, mllt4, ipo13, zgc:158605, zmp:0000000679, gatm, znf292b, nktr, sipa1l1, appbp2, metap1, si:dkey-83k24.4, ahcyl2, farsa, ctu2, slc1a5, st5, zbtb17, herc3, itsn1, arhgef1a, prpsap2, ppp1r7, s35e2, loc101161630, slc35b2, dhx34, psmd7, ccnyl1, appl1, cadm4, rnf20, zgc:113006, shmt1, nub1, mettl14, flt4, wrnip1, ing3, b3glt, coro1b, cdc40, vwf, cnot7, cryz, znf148, hipk3a, cdk8, map2k1, bcl9, tpst1l, bdh2, c1d, kif21a, napa, sbf2, ppan, pds5b, ulk3, ercc3, golgb1, coro1ca, mfsd2ab, trim33, pex10, cxxc1, lphn3, hipk3b, loc101171877, sec23b, lrp12, amotl2a, med31, fam65a, sart1, zgc:91940, rund1, ano5b, kmt2c, syncripl, elp2, alg12, ppp2ca, si:dkey-250d21.1, usp47, loc101170986, loc101158698, wdr41, polr2l, c1galt1b, cactin, kbtbd4, dbt, si:dkey-60b12.1, slc38a7, traf3ip1, sema6dl, fancd2, yipf5, dynll2, tmem131, rab43, zgc:101777, kctd7, cox7b, kdm2ba, myo5aa, foxred1, hiat1a, loc101162428, xdh, chch5, opa3, zgc:77650, stx16, mpv17, bfar, osbp, plekha5, loc565999, klhdc10, acad8, mfn2, plxnb2a, ptpn13, bach1, pik3c2a, decr2, mcm4, edem1, cobl, fsd1l, ubr2, stxb3, qpctla, micu2, cbwd, aspa, camkk1, loc101175285, slc20a2, pimt, arid4a, fpgs, fam193b, bcat1, slc31a1, vac14, cebpa, zgc:175171, cpne8, dbf4, tes, dc1i2, taf5l, asap2a, loc100695856, cep135, klc1, rnf44, dst, thap1, txnl1, schip1, ddx49, e4f1, loc799686, pex13, zc3hc1, tcea3, gpn3, capn5a, dock4b, ap3s1, ube3b, mtmr2, mon2, aarsd1, tlk2, dapk1, prdm4, rps6kc1, mpp5a, slc47a2, bivm, ercc8, ifrd2, erlec1, ptpn21, nutf2, ptprc, loc101077062, mad1l1, loc101068170, wbp1la, zeb2b, sec31a, lrp5, wdr46, dapk2, strumpellin, pomt2, klc1b, mark1, abtb1, cux1, zbtb1, acbd6, slc7a7, mlycd, arhgef11, tbl3, sylc, kdm4b, top3a, pnkd, cs060, magi1b, si:ch211-152c12.2, bbip1, arl6ip4, pofut1, lactb, loc100710089, smpd1, sass6, ptbp2, qser1, ptgesl, rtel1, smyd2a, inppl1a, mfs11, cfl2, esyt1a, loc101172694, hint1, jph2, si:ch211-89f7.1, pofut2, lmf2a, dhdds, nedd4l, stk4, lrpap1, tmem63a, hal, gmfb, ehd1b, phgdh, uevld, si:dkey-119o24.1, srsf5a, tm9s3, loc100699426, vldlr, notch2, fbxl18, nphp3, tmem161b, trim24, slc25a14, iqcc, topbp1, ikbke, loc100696751, med12, acot7, dia1b, ehd2, col5a1, lcor, cpvl, loc101077393, fmr1, ptk2.2, mb21d2, adck1, scly, ano1, vrk3, btbd9, kpna2, setd6, atg4c, slc25a40, pcgf1, ofd1, loc101073674, mocs3, loc101170954, rnasen, csrp2, lrrc1, gm15800, dhx30, phka1, vcpip1, march7, slc44a1, sox7, tmub1, dak, al7a1, fras1, eml2, ap5m1, bckdk, rap2a, naca, rnf145b, fam98a, disc1, mief2, bin1, loc101070651, dlg1, dync1li1, cdc123, znt6, ppm1e, git2b, atg5, mta1, slit3, usp24, smap2, tiprl, zgc:73185, sec22bb, mdn1, casp8, smek1, rnf11a, ctbp1, med27, fut11, vdac2, kirrela, dgke, slc12a4, focad, pebp1, fbxo30a, tbcd, rab7, ddx59, zc3h15, rcc2, loc100710887, scrib, cnot6, tldc1, poli, samd11, sec62, tsc1, rfng, abcc8, kif1c, pygb, si:dkey-5g7.3, noc4l, loc101175197, zgc:113278, loc101078366, gpc1b, pex7, cpeb2, dnmt1, cyhr1, bola1, fgfr1a, polk, wdr55, kat6a, loc101169385, atxn2, zgc:194800, adam9, pias4a, inpp4ab, myeov2, zbtb18, fbxo31, loc100695128, loc100194675, trim36, cnn3b, iqgap1, jmy, loc101156767, kiaa0947l, tle2, tmem55a, wdr37, rps26, zgc:162898, psmd1, sorl1, plbl1, loc100701644, si:dkey-110k5.6, si:dkey-12e7.1, mre11a, pdia3, icef1, rrs1, atad1a, gsk3ab, stam, cpeb4, snx11, sptssa, atp2b4, si:dkey-33c12.4, ctc1, larp4b, vav1, epb41l5, prickle2, znf592, parp16, atxn3, comd4, mus81, tmed2, nudcd3, tbc1d13, loc100699648, pecr, gsk3b, pigk, slc7a2, snrpc, nr1d2b, pip5k1ab, banp, dpy19l1l, acer2, ttll11, rangap1a, zgc:175140, papl, rnf126, plcb3, dcam, sulf2l, loc100002601, zfyve9, letm2, slc17a9b, tha1, e41l2, btbd2, m3k8, btk, setdb1b, spopla, ptrfb, zgc:65772, loc100699974, slc27a4, loc100700120, pold2, sema6e, loc101171294, slc46a1, kcnk6, ankrd44, loc101067285, ddx17, herpud2, gk5, tcf4, ogg1, drosha, exoc6b, tdp2b, enpp1, znf395, rnf13, sh3gl1b, znf598, clock, rgs12b, dmxl1, hebp2, brd9, supt7l, hells, mfsd5, rad23ab, edem2, rn121, palld, loc101169137, smchd1, mospd1, nlk1, smoc1, mapk9, zgc:194562, dvl1b, tnc, loc101169512, inip, coq10b, ncoa1, nd4, zranb2, zgpat, galc, wwp2, kiaa0100, cdkl5, sin3aa, wdr45l, tmem86a, si:dkey-103i16.1, smoc2, acss1, vcam1, wdsub1, sash1a, loc101062201, cenpi, chmp4b, loc101071947, plk3, eif4enif1, tnks, loc101173440, zgc:56556, thoc7, anp32a, galnt2, cap2, ripk2, aldh9a1b, gan, gas7, paf1, cyp27a7, si:dkey-275b16.2, smarcc1a, olfml2a, rfc2, vegfc, fyb, tspan5a, zgc:153972, tmem115, pi4k2b, loc101167691, dmbt1, cdk5, loc100702488, irs2, slc33a1, c16orf52-b, zfhx4, cebpg, rnf111, psmg1, loc101069981, nol12, bicd2, pter, loc100002393, lsmd1, eps15, hook1, cep76, unc119.1, zgc:100829, mfap1, tmem161a, loc100699751, dnajc27, addb, zc2hc1a, zgc:172180, star5, gpd1l, ppp1r14bb, l2hgdh, loc570835, palm2, srd5a3, tenm3, wee1, loc100711569, rbm14b, asphd2, loc101171436, txnrd2, si:dkey-1h24.3, loc100697442, si:zfos-452g4.1, tmem35, armc9, chchd3, kal1.1, nek7, atp6v0a1a, loc101155212, wdr75, slc37a4b, nans, grb10a, tm9sf1, bgnb, rgma, zgc:153901, cd226, metrnl, flr, gga3, brf2, dub, git2, ptprf, loc100005105, cnot3a, cab39l1, chac2, olfml3b, pus3, slc35a1, vamp4, loc100698144, yars2, zgc:91845, dph1, nbeal2, cbx4, eif3i, sepw2a, fbp1a, gorasp1, ppih, plxna1, lgmn, accs, trmt2b, loc100705078, pgk1, bcam, zgc:153225, rnf115, tmem8a, tubgcp4, cbfb, selu, lrrc59, ccs, trak1, klf11b, itga2b, neurl4, si:dkey-204a24.9, acd, pesc, rabgef1, slc25a29, anxa13, ptprub, lin9, zgc:66014, pcnp, trmt44, clcn6, tead1, tprkb, chico, plcg2, rasl12, vezf1b, dab2ipb, sufu, blm, pbx2, loc100703307, dyrk2, plxna3, rab3ip, si:dkey-204f11.59, npl, loc101167776, zgc:158403, kpna5, ube2f, wash, aak1, eng1b, lama2, rev1, slc35b1, hmgn3, rnf2, pdhx, tmem104, dlg2, ercc1, loc101061707, thbs1a, tbrg4, ari2, zn410, alg1, ikzf5, wbscr22, zgc:136929, kiaa0261, glt8d1, loc101156868, prdm11, rps6ka3a, tacc2, atp8a1, megf10, loc100000994, parp12b, dclre1c, slc44a5b, dph5, klf12b, rab11fip4a, brd3a, ercc2, otud3, akap1b, rpc8, spire2, wu:fj20e02, cpeb3, ppp2r5a, cgrrf1, rn181, smc2, efnb1, slc35b3, srfbp1, cpne3, daxx, tacc3, fip1l1b, gnai2, slc4a2b, g3bp1, grk6, rhbdd1, ergic2, akap2, uroc1, edc3, sfrs3b, aes, csn4, gatsl2, xkrx, lmo2, polm, dazap1, lrrc40, abcd2, traf3, atrip,

i2b2a, whsc1, pstpip1a, cct3, shdb, actr5, sp2, prkci, leg8, klh13, loc100706979, si:dkeyp-113d7.1, pdzd8, dus1l, loc100692840, esrra, stam2, atg12, etfb, trim71, loc100706158, gpr137ba, s38a2, bcas2, sae1, si:ch73-248e21.1, si:ch211-193c2.2, pan3, ubqln4, pde4b, sh3rf1, fitm2, creb3l2, adamts10, loc101073420, ddx11, zgc:158343, crlf3, loc100708388, hps1, dnajc7, fam160b2, loc101158462, synpo, man2c1, loc101170991, loc100700218, bcl2l1, ubxn6, anapc1, sp30l, arcn1, dcun1d4, dmtn, fbli1, ppcs, zgc:55573, golph3, klhl24, rorcb, mrps31, oxnad1, ppl, intu, mgat1b, ppp1r13bb, aldh8a1, usp13, nrbf2, loc101065336, pgbd5, zeb1b, fgfr3, arpc1a, hadhb, ct004, phf12, si:dkey-102c8.6, srpk3, kbtbd8, taf5, vegfaa, pou6f1, loc101078156, tmed5, cc032, cep63, nosip, si:ch1073-357b18.4, adcy2b, trim13, plxnb2b, msmo1, ebp, usb1, hs2st1, stat3, prs6a, josd2, ralgps1, cdr2l, loc100693258, ileu, si:ch211-208m1.2, ficd, prep, mical1, loc101159889, trappc13, zgc:113070, cep68, dcup, nudt14, brdt, zgc:55733, kcnma1, fndc3ba, akap8l, atad3b, akt2, elk1, zgc:158437, cnksr1, snrkb, hgfa, igsf11, aldh9a1a.1, lpp, loc101068251, wdfy3, zeb2a, sec14l1, sytl4, h2afy, pde10a, katnb1, pomt1, znf609, prkca, cuedc1b, fbln2, mex3b, akap9, atf5a, pik3ca, arhgef3l, prr5, zgc:172359, zdh13, slmapa, loc100698890, znf207a, tmem30ab, armc6, hlcs, eftud1, nsma3, gmeb1, ilf3, cyp4v8, meis2, agpat2, mepd, loc101158963, atxn7l3, ssr1, leng9, adat1, wu:fb39e10, loc100707082, tnni3k, prp19, sertad2b, eaf1, p4ha1b, zgc:103657, si:ch211-42i9.8, etnppl, tcpg, fry, kiaa0586, pole2, enpp5, zgc:153169, rfc3, tube1, tsen54, uchl3, nt5c2b, yap1, loc100694533, agpat3, gde1, postnb, fancm, ier3ip1, mk67i, fam105ba, foxj2, zgc:101846, cnot2, scube3, sntb1, trmt13, zgc:152863, apc7, bpnt1, psmd3, mpp6b, im:7137555, pdgfrb, kcnd3, tango2, tfg, faah2a, tnfaip3, gfer, faf2, pbrm1l, txlng, spcs1, akap10, cmtr2, si:dkeyp-55f12.3, hpcal4, staf, p2rx7, rpgrip1l, ngap, si:dkey-230p4.1, alox5, gys1, loc100698001, dhx29, med24, abcc2, ccnt1, kif19, skia, smpd2a, agk, sox14, loc100692366, mesd1, loc101169532, loc100535895, loc101157224, arhgap39, cflar, loc101163156, rxrba, loc101079712, loc100690556, cyb5b, hsd11b3a, boc, csnk2a1, zfand1, selt2, loc564844, ctnna2, si:dkey-6n6.7, loc101068474, loc101075369, loc100701951, si:dkey-177p2.6, ccbl1, ptk2bb, prmt6, crfb2, brpf3, loc101078965, slc7a1, herc1, ipmkb, loc568935, ech1, tiam1, hhatla, si:dkey-260j18.2, nit1, syt4, wdr12, rprd2a, loc101077847, atp5h, xpnpep3, rps6ka1, rasef, tmm47, loc100700477, si:ch211-218c6.8, loc101158931, abhd17c, sb:cb54, hdac5, rnh2b, nes, flrt3, tmeff2a, hdr, slc17a5, loc101173334, ric8a, trim62, zcchc24, loc100695413, zgc:66440, loc101172779, smarce1, loc101162589, loc100710191, egfra, loc101161733, derl1, stx5a, acta2, zgc:91860, imp4, loc100695978, loc566292, cops5, gab2, loc101173335, ghdc, unk, rhog, arhgap42a, kmo, loc101079008, loc101061856, galnt1, hexim1, mcl1b, atox1, jmjd7, mtfmt, pgam1a, trmt5, cl045, stk3, tmem39a, loc101173792, renr, nrp2a, stra13, bcl7ba, tacc1b, pde2a, wdr81, zgc:153351, smpdl3a, wfs1, pop5, loc101161900, hnrnpd, loc100701830, loc100691261, cd276, si:ch211-238n5.4, adam8a, pdrg1, if3ei, zgc:56041, rtn1b, ankrd6b, sphkap, alas2, hey1, rccd1, snx21, tp53bp2, ube2r2, sgms1, hm:gc12, ce044, nsd1a, rft1, si:ch211-214j24.10, tm39b, ehhadh, bet1l, actr3, angpt1, bhlhe40, hnrnph1, tmx4, commd9, loc101160056, rbm41, dysf, st6gal1, vps4a, tsr3, zswim6, loc101075621, hs6st2, gxylt2, coa3, sdsl, nebl, sptlc3, zgc:162344, loc101158689, pdcd6, sap130a, zgc:77415, f11r, si:ch211-214j24.7, sema6a, f5, loc101065618, comd1, mettl16, copt2, nudt4a, rab35, loc101073416, loc101076929, acap3b, ci142, rufy3, ndrg4, foxn2b, loc101065228, loc100708649, si:dkey-24p1.1, alkbh2, zgc:110289, armc1, psma1, fam222b, efnb2a, strap, apmap, tsnax, clip3, tfcp2, zgc:165666, crb3a, loc101063663, loc101163857, pbxip1b, assy, vps26a, cetp, arhgap26, loc798528, zgc:63520, nfs1, ttc14, prr14, loc100695307, lpar1, spred2b, emp2, loc101163435, zgc:112083, pxmp2, apoer2, mprip, kif7, loc100701761, angptl1, vgll4b, prkar1b, eral, loc101170515, otud5a, zgc:56493, si:dkeyp-69c1.6, zgc:158420, loc100332331, rchy1, loc100709720, slc25a35, pmvk, frem3, loc100705008, nras, zgc:110329, si:dkey-33i11.3, kif23, f262, i2bp1, slc13a2, pdlim4, znf740, si:dkey-97o5.1, rhbdf1, b4galt4, klhl42, errfi1, zgc:103559, setdb2, mmrn2a, elp4, tor2a, mthfd1, snx27a, ap3m1, trnau1apb, psd3, cyp26b1, n4bp2, zgc:110655, loc100702655, sin1, pomk, ctns, si:ch211-222g5.3, pdik1l, sepp1b, dpm3, zfpm1, grsf1, inpp5e, mrps33, antxr2a, ttc19, atg16l1, dnjc7, zgc:109744, ptrh1, snx15, tcf7, baz2ba, cdk20, paip2b, atp1b3b, s39ab, mtmr1b, arpc1b, polr1c, chd3, si:ch211-199g17.1, caza1, loc101077335, im:7136185, abhd11, ehmt1a, phtf2, crbn, loc101067958, sergef, acbd5a, ptpn18, serpini1, slc7a6, npnt, tnrc18, zwi, adka, aldh18a1, ripk1l, hsbp1b, rl7l, loc100691923, ralgps2, loc100703867, sgf29, loc101062035, bpgm, si:ch211-195b13.1, mospd2, apitd1, med20, gmds, tm7sf2, park2, loc101167393, ssb, agtrap, cbx1a, rsad1, sp3a, dvl2, cc060, loc101078232, esama, loc100698701, tmem206, mntb, slc25a6, sdhdb, aff1, tmem47, loc101078597, enpp4, ftr14, tmem59l, loc100701553, nalcn, loc565251, prkcb, faah, fzd7a, slc16a6b, ssu72l, cers5, si:dkey-184p18.2, npas2, atad5a, loc100691521, dnajc25, loc101161328, atg2b, trim47, zgc:65870, irf2bp2a, ube3d, fam217b, pgrmc1, loc562940, si:ch211-199o1.5, mphosph6, si:ch211-210c8.6, skap2, actn3b, fyco1, loc100699414, camk2a, loc100700824, itih2, npr1a, rims2, dnali1, sez6l2, fra10ac1, lppr2a, loc100693156, ppp6r3, nek6, chd5, loc101078699, epha2, loc100329955, scai, loc101169712, cox5b2, si:dkey-217d24.6, wasb, pnn, loc101078463, irx1a, mdm1, tktb, zgc:110779, loc100693225, zgc:77262, ilf3b, znf507, lrrc57, ufc1, loc100000800, cbx3a, commd3, zfx, mybpc1, dnajb11, wu:fb54a03, loc101073444, mblac2, loc101169718, ptpn2a, lin7a, si:dkey-231l1.3, gpr125, dpysl2b, hint3, rm38, psf2, phb2, map4k3, si:dkey-109j17.5, loc100711401, abcg2, tfip11, mrpl30, ptgr1, loc100690208, si:ch211-247j9.1, mfap4, eif4g2b, txtp, btbd1, ndufa4, zgc:66433, fstl1b, zgc:77838, fam78ab, im:7138535, lrrk1, mtus1a, tagapb, b3gat1, cmya5, loc100701090, arhgef9b, loxl2b, aebp2, aagab, olezh1, rprd2b, tlcd2, gbf1, psmb3, fglh1, fam45a, loc101065472, ccnt2b, rnf146, znf513, si:dkey-10o6.2, skilb, nol7, slc16a12b, loc100698582, dusp12, stag2a, etaa1, loc100537934, loc100333416, pnrc2, dok2, loc571530, amn1, xrg5, si:ch211-214e3.5, loc101156156, pde8b, grb2b, kif5aa, ctnnb1, prkag2, arap3, tgfbr3, mkks, arhb, rbm4.3, si:ch211-105d11.2, ppil3, zgc:101744, crym, loc100707314, entpd1, rgcc, i20l1, eif1ad, loc565514, dacha, lsm14ab, gemin2, zgc:56072, kcnq3, rwdd, fam160a1, ciz1b, loc100140695, myo1f, zdhhc16a, fga, loc101165234, hvcn1, smpx, zgc:163143, tal1, hcn1, loc100535018, ahnak, tfpt, ncbp2, mxi1, loc100706969, smu1, ankrd37, klhl40a, wu:fc51b03, nemvedraft_v1g210604, hif1al2, ogfr, zdhhc8b, jund, aftph, loc101079231, loc100712470, morn4, slc30a7, atp5o, loc100690900, loc101075289, mtx1b, loc100703264, vsnl1a, ebi3, loc100708546, cxxc1l, nhsl1b, stc2a,

	timelessa, loc100695375, dr1, syt11a, stip1, pdap1b, loc101068319, atp6v1aa, si:ch211-214j8.1, ctnnd2, cbsa, kith, loc100698311, r3hdm4, aven, cu070, pc, loc101172487, dusp2, uros, gtf2h2, emd, gpr137bb, arl14ep, alkbh4, zeb1, sgcd, fsd1, loc101166169, kiaa1033, zgc:110843, loc101071133, c1galt1c1, loc793850, loc101079356, napg, stxb2, shf, yjefn3, abr, serbp1a, ptn6, scg3, fam120c, rundc3ab, slc7a4, fam135a, kmt2e, zgc:63568, loc100194683, ebna1bp2, stea4, slc27a6, gpr18, loc100690798, utp20, kiaa1841, dhrsx, ezh1, gchfr, lcat, loc100699244, lrtm2, loc100693554, si:dkey-159f12.3, sspn, gabbr2, astn1, rhoab, rtn4a, loc100194603, rundc3b, loc100711919, loc101071480, hgd, tcea2, cntnap1, ublcp1, znf330, med19b, ctds1, actn2, zgc:153953, ywhaqb, loc100701990, spata5l1, si:dkey-199f5.8, igfbp-rp1, dnal1, jakmip3, stk16, manea, rap1a, loc101161715, tsku, clic2, loc100706661, dlc, zgc:92360, gabpb1, adcy3l, trim16, zbtb3, cox4i2, rcn3, cpb2, ndufa10, lrch4, loc100693265, loc101166285, slc37a3, cpsf7, loc101076009, loc100703115, tardbpl, loc796628, eif4a3, unc79, arhgef6, loc101173566, scube2, dennd4a, loc100708681, loc100711013, agap1, samd9l, gna11b, pon2, tspan12, parva, zgc:153981, slc16a7, ppifa, loc100696163, si:ch211-254n4.3, loc101166923, wbp2, zgc:66484, ppp3cb, ramp3, loc100700405, slc37a4a, vegfr, dpf2, mybl2, loc100704509, dusp6, rad51b, enah, chac1, loc101075798, loc793421, mapk15, srsf5b, nbeaa, pp25, si:dkey-91i10.2, cc107, loc101078884, ror1, ncoa7, b3gntl1, u2af1, loc100004452, loc570267, loc100712301, zgc:162316, zgc:175274, vasnb, loc101164549, hbegfa, wfdc1, slaf7, loc100708009, adck2, loc567924, loc101065171, tmem194b, loc100537899, cygb1, loc100693079, loc101174720, bcl6ab, loc101163716, loc101073979, loc796135, mxd1, adam7, azi2, camk2d1, acaa1, grnb, ptk6b, u2af2a, zgc:64098, aox1, zgc:173816, si:ch73-308m11.1, ts101, f108b, nlrx1, loc100000155, appb, zfp532, loc101161104, loc100698437, hecw1, ergi2, cdc14a, tnw, atf7b, ccdc151, tomm34, rit1, loc100695366, usp43a, efcab7, hmgb1a, loc101076635, sarnp, pss, col6a1, ablim1, zgc:113121, rsph3, ncaph, loc100696046, brinp1, loc101070739, loc101072715, loc101160316, ppp1r18, loc101166009, loc100698866, khdrbs1a, si:dkey-281i8.1, exosc10, znf385b, chek1, mpped2a, atraid, rgd1560328, mre11, gria2, loc100699645, cox17, si:dkeyp-68b7.5, loc101168880, banf1, loc100703649, loc100704905, stim2b, pcnx, loc101070379, loc101158623, rps10, socs5, si:dkey-33c12.3, loc100706632, traf2a, lcris_00442, loc101070000, comd2, loc101067948, loc558232, loc100304587, lysmd3, cry-dash, spon2b, ck068, 2310067b10rik, nrsn1, fitm1, klf15, gnpi1, loc101073417, loc101077580, st6gal2a, cx43.4, ryr1, gtpbp1, nr2f5, ap3b2, hmbox1a, loc100698106, ago3, pdgfaa, clip4, mrpl19, mimit, fbxo33, loc101174474, cxxc4, arid3a, loc100703671, loc100690694, zdhhc18b, ca085, spsb3a, map3k3, zgc:193801, loc101160998, loc100711612, ppiab, nduc2, loc101165038, carkd, loc100693100, caskin2, loc100695530, loc100689760, kif26a, orml1, yk001, loc101867533, fa82b, loc101072596, si:dkey-197c15.6, loc100640810, spsb4b, loc792383, zgc:194281, loc564666, cahz, chmp3, loc100705661, loc100535085, tmem154, zgc:158357, uqcrc2b, loc101075637, stat4, acot11a, col1a2, agfg1a, tr112, loc100704154, mtss1, prkab1a, muc2, cers4a, spata6, elovl1b, trim46b, loc100708573, timm17b, arl16, hsd20b2, tm187, plcd3a, loc101070602, yes1, zgc:86841, ykt6, loc101161181, zgc:56576, fam168a, loc101062525, pmm2, dnm1a, ck2b, mcph1, dpyl3, zgc:162184, loc100712285, loc100690803, fam53b, cldnk, abi1b, loc101161944
***Euteleostei* (85)**	*yars, cars, trip12, rab3gap2, herc2, vwa8, nup98, nfyc, dync1h1, ddx54, skiv2l, thoc2, parp3, ankhd1, snx14, csnk1a1, zw10, usp34, aktip, rtf1, exosc1, xpo4, atrn, si:ch211-233a24.2, clk2a, ogt.1, narfl, acadvl, ap4m1, eif4g2a, nxf1, ahcy, slc25a42, pus7, abcc4, clip1, tufm, spag1a, alg11, bcl9l, ik, clasp2, ube2z, thoc5, rab10, usp36, phf8, pld3, sptbn1, srek1, chd8, zc3h14, nt5dc2, plp2, ap3s2, usp38, usp40, extl3, cwf19l1, pdcd7, nupl2, gpatch1, sfpq, mbnl1, med13b, ero1lb, ccdc174, si:ch211-93f2.1, if2b, cables1, loc101071127, cpsf3, mettl25, zgc:172112, pkp2, mrps34, im:7151068, gas8, march5l, si:dkeyp-110c7.1, zcchc9, dnmt3a, unc5c, stx17, ppp3ca*
***Actinopterygii* (19)**	*yars, cars, trip12, rab3gap2, herc2, nup98, nfyc, dync1h1, ddx54, skiv2l, thoc2, snx14, csnk1a1, zw10, xpo4, atrn, si:ch211-233a24.2, ogt.1, narfl*
***Chordate* (8)**	*yars, trip12, nfyc, ddx54, skiv2l, thoc2, snx14, zw10*
